# Nitric oxide regulated cyclic di-GMP signaling

**DOI:** 10.1186/2050-6511-14-S1-O37

**Published:** 2013-08-29

**Authors:** Elizabeth M Boon, Zhou Dai, Niu Liu, Bernadette Henares, Dhruv Arora, Tanaya Lahiri

**Affiliations:** 1 Department of Chemistry, Stony Brook University, Stony Brook, NY 11794-3400, USA

## Background

Nitric oxide (NO), a well-known signaling molecule in eukaryotic organisms, has recently been implicated in the development of bacterial biofilms. Biofilms are surface-bound, matrix-encapsulated, multicellular communities that are extremely resistant to antibiotic treatments. In addition to being important in many aquatic, industrial, and environmental processes, biofilms are responsible for ~60% of all human non-viral infections. NO has been reported to disperse bacterial biofilms through regulation of intracellular cyclic-di-guanosine monophosphate (c-di-GMP) concentrations. C-di-GMP is a tightly regulated second messenger signaling molecule that is tightly correlated with biofilm formation. When we began our work, however, the NO sensor(s) and biochemical signalling pathway(s) that regulate c-di-GMP concentrations was unknown.

## Results

H-NOX domains are evolutionarily conserved heme proteins that include the well-characterized eukaryotic NO sensor, soluble guanylate cyclase (sGC). In the genomes of many bacteria, an H-NOX gene is found near a predicted diguanylate cyclase and/or phosphodiesterase gene. These two classes of enzymes are responsible for regulating the intracellular concentration of c-di-GMP. Therefore, we hypothesized that H-NOX may sense NO and control the enzymatic synthesis or hydrolysis of c-di-GMP, providing a molecular explanation for NO-mediated biofilm dispersal. Indeed, we have shown that H-NOX is a modular NO sensor that negatively affects biofilm formation through one of several mechanisms. Generally, H-NOX regulates c-di-GMP processing enzymes, either directly or through an intervening histidine kinase. In *Vibrio*, NO-bound H-NOX can enter quorum-sensing circuits through a histidine kinase, which ultimately also mediates biofilm formation.

We have also pursued understanding the molecular details of this pathway. NO binds H-NOX at the iron atom of a histidine-ligated heme cofactor that is severely distorted from planarity. It has long been assumed that histidine dissociation upon NO ligation is the critical step for signal initiation. However, we established that the iron-histidine bond is retained after NO binding. Instead, we demonstrated that heme flattening upon NO binding is the trigger for signal transduction. As H-NOX proteins are bacterial homologs of sGC, we expect the initial signal transduction steps in sGC are similar.

## Conclusion

We have identified molecular mechanisms for NO regulation of bacterial communities. This work is the starting point for deeper investigations into the role of NO in bacteria and bacterial/host interactions. These fundamental investigations will result in novel strategies for biofilm regulation with widespread application in medicine and industry.

